# The effects of different acupuncture modalities on postoperative cognitive function in elderly Chinese patients undergoing general anesthesia: a network meta-analysis

**DOI:** 10.3389/fneur.2025.1637566

**Published:** 2025-09-19

**Authors:** Wenjie Liang, Mengzhong Li, Jianguo Zhang, Wei Liang

**Affiliations:** ^1^Ruikang Clinical Medical College, Guangxi University of Chinese Medicine, Nanning, Guangxi, China; ^2^Acupuncture Department, Ruikang Hospital Affiliated with Guangxi University of Chinese Medicine, Nanning, Guangxi, China

**Keywords:** acupuncture, network meta-analysis, Mini-Mental State Examination, postoperative cognitive dysfunction, electroacupuncture

## Abstract

**Background:**

Postoperative cognitive dysfunction (POCD) is a syndrome characterized by long-term cognitive impairment following anesthesia and surgery. Acupuncture has demonstrated potential therapeutic benefits in managing POCD. However, comparative efficacy among different acupuncture modalities remains unexplored. This study aims to systematically compare the effects of various acupuncture interventions on postoperative cognitive function in elderly patients undergoing general anesthesia.

**Methods:**

A comprehensive literature search was conducted across eight databases—CNKI, Wanfang, VIP, SinoMed, PubMed, Embase, Cochrane Library, and Web of Science—up to January 2025. Randomized controlled trials (RCTs) assessing acupuncture interventions for POCD in elderly patients receiving general anesthesia were included, provided cognitive outcomes were measured by the Mini-Mental State Examination (MMSE) or reported POCD incidence. Study quality was appraised using the Cochrane Risk of Bias Tool 2.0. A Bayesian network meta-analysis (NMA) was performed with the GEMTC package in R software, incorporating both direct and indirect comparisons. Intervention rankings were evaluated using the Surface Under the Cumulative Ranking Curve (SUCRA). Statistical significance was set at *p* < 0.05. Publication bias was assessed by funnel plots generated in Stata 18.0.

**Results:**

Thirty-two studies involving 2,644 patients were included. The SUCRA rankings for efficacy in improving postoperative cognitive function were: Electroacupuncture (77.93%) > Thumbtack Needle (73.89%) > Scalp Acupuncture (68.58%). Subgroup analysis by intervention timing revealed: preoperative phase—electroacupuncture was significantly superior to conventional anesthesia and thumbtack needle; intraoperative phase—electroacupuncture outperformed scalp acupuncture and placebo; postoperative phase—electroacupuncture showed the best efficacy, surpassing conventional anesthesia and Xingnao Kaiqiao acupuncture; perioperative phase—auricular acupuncture exhibited notable advantages over electroacupuncture and standard of care. Regarding POCD incidence, 23 studies with 1,886 patients demonstrated SUCRA rankings as: Xingnao Kaiqiao acupuncture (86.56%) > Thumbtack Needle (80.16%) > Electroacupuncture (58.78%).

**Conclusion:**

Electroacupuncture exerted the most substantial effect in mitigating postoperative declines in Mini-Mental State Examination (MMSE) scores among elderly Chinese patients receiving general anesthesia. Thumbtack needle acupuncture and scalp acupuncture also showed relatively favorable benefits. Electroacupuncture consistently achieved superior outcomes across preoperative, intraoperative, and postoperative interventions.

**Systematic review registration:**

https://www.crd.york.ac.uk/PROSPERO/view/CRD420251061472.

## Introduction

Postoperative cognitive dysfunction (POCD) is a central nervous system complication following anesthesia and surgery, characterized by impairments in memory, attention, language, and orientation (1, 2). The elderly population is particularly vulnerable to POCD (3), with incidence rates in non-cardiac surgery patients aged over 60 being 1.5 times higher than those in younger individuals (4). Hospital prevalence among older adults can reach up to 41.4% (4, 5). POCD may persist from several weeks to years, prolonging recovery time and hospitalization, while increasing mortality and the risk of psychosomatic comorbidities (6). With accelerating population aging in China, the increasing number of elderly surgical patients imposes a growing burden of POCD on families and society.

Current prevention and management strategies for POCD focus on three aspects: optimization of anesthesia protocols (e.g., total intravenous anesthesia with propofol), pharmacological interventions (e.g., dexmedetomidine, lidocaine, parecoxib), and non-pharmacological approaches (e.g., preoperative cognitive training and acupuncture). However, challenges remain due to incomplete elucidation of POCD pathogenesis, lack of evidence-based standards for individualized anesthesia, inconsistent drug efficacy, and limited clinical evidence supporting non-pharmacological interventions such as acupuncture and rehabilitation strategies (7, 8).

Among existing evidence, only one meta-analysis has addressed acupuncture intervention effects on POCD (9). Although it confirmed acupuncture significantly reduces early postoperative cognitive impairment in elderly patients, the analysis was limited by a narrow focus on specific acupoints and failed to systematically clarify efficacy heterogeneity among different acupuncture modalities. Traditional pairwise meta-analyses face methodological constraints when comparing multiple interventions simultaneously, limiting multidimensional efficacy assessment. Therefore, this study employed, for the first time, a network meta-analysis approach integrating direct and indirect evidence to comprehensively compare and rank the therapeutic effects of different acupuncture interventions on POCD. The aim is to provide evidence-based guidance for optimizing acupuncture strategies in the perioperative management of elderly patients.

## Methods

This study was conducted in accordance with the Preferred Reporting Items for Systematic Reviews and Meta-Analyses (PRISMA) guidelines, including extensions for network meta-analysis (NMA) (10). The study protocol was prospectively registered on the International Prospective Register of Systematic Reviews (PROSPERO) under the registration number CRD420250651273.

### Search strategy

A comprehensive literature search was performed across eight databases: PubMed, Embase, Cochrane Library, Web of Science, China National Knowledge Infrastructure (CNKI), Wanfang Data, VIP Database, and SinoMed. The search period spanned from the inception of each database to January 23, 2025. Language restrictions were set to English or Chinese. The search combined Medical Subject Headings (MeSH) and free-text terms related to Acupuncture Therapy, Electroacupuncture, Moxibustion, and Postoperative Cognitive Complications. Additionally, reference lists of relevant articles and gray literature were manually screened to identify further eligible studies. The detailed search strategy is provided in Appendix 1.

### Inclusion and exclusion criteria

Studies were included if they met the following criteria: (1) Population: Elderly patients aged 60 years or older undergoing surgery under general anesthesia, regardless of sex; (2) Interventions: Electroacupuncture, Scalp Acupuncture, Ear Acupuncture, Acupuncture, Thumbtack Needle, Xingnao Kaiqiao Acupuncture; Comparators: General Anesthesia, Standard of Care (SoC), Placebo; (3) Study design: Randomized controlled trials (RCTs); (4) Outcomes: MMSE score (change from baseline to postoperative assessment) and incidence of postoperative cognitive dysfunction (POCD); (5) Language: Chinese or English.

Exclusion criteria were as follows: (1) animal or cell studies, case reports, study protocols, reviews, letters, editorials, conference abstracts; (2) studies with missing or erroneous data; (3) duplicate publications; (4) unavailable full texts; (5) overlapping participant data in multiple publications; (6) elderly patients with pre-existing neuropsychiatric disorders undergoing general anesthesia.

### Data extraction

All retrieved records were imported into EndNote. Two independent investigators (Liang and Li) screened titles and abstracts according to the inclusion and exclusion criteria, followed by full-text review for final eligibility assessment. Discrepancies were resolved through discussion or consultation with a third investigator (Zhang). Data extraction was performed independently by two investigators using a pre-designed electronic form, including first author, publication year, country, sample size, patient age, intervention and comparator details, treatment duration, and outcome measures.

### Quality assessment

The risk of bias of included RCTs was independently assessed by two authors (Liang and Li) using the Cochrane Risk of Bias tool version 2.0 (ROB2.0) (11). The ROB2.0 evaluates five domains: random sequence generation, allocation concealment, blinding of participants and personnel, incomplete outcome data, and selective reporting. Each domain was rated as “high risk,” “some concerns,” or “low risk.” Overall study risk of bias was classified as low risk if all domains were low risk or only one domain had some concerns; high risk if four or more domains had some concerns or any domain was high risk; and moderate risk for other cases. Any disagreements were resolved by the third author (Zhang).

### Statistical analysis

Continuous outcomes were analyzed using mean differences (MD) with 95% credible intervals as effect measures. A Bayesian network meta-analysis model was constructed using Markov Chain Monte Carlo (MCMC) simulation implemented in the GeMTC package in R. Model parameters included four chains, 10,000 burn-in iterations, 100,000 sampling iterations with a thinning interval of 10, and initial values set to 2.5, aiming to obtain posterior distributions. Three core assumptions underpinning network meta-analysis—transitivity, homogeneity, and consistency—were evaluated. Heterogeneity was assessed using the mtc. anohe function in GeMTC; an overall *I*^2^ < 50% was considered acceptable to satisfy the homogeneity assumption. Consistency between direct and indirect evidence was examined by the node-splitting method (mtc.nodesplit function); *p*-values > 0.05 indicated no significant inconsistency. Convergence was assessed by calculating the Potential Scale Reduction Factor (PSRF), with a value close to 1 indicating satisfactory convergence. Network geometry was visualized with nodes representing different interventions and edges representing head-to-head comparisons. Placebo served as the reference comparator for all analyses. The Surface Under the Cumulative Ranking curve (SUCRA) was calculated to rank the interventions. Publication bias was assessed using funnel plots. All statistical analyses were conducted using R software version 4.4.1 and Stata version 18.0.

## Results

### Literature search and screening process

**Figure 1:**
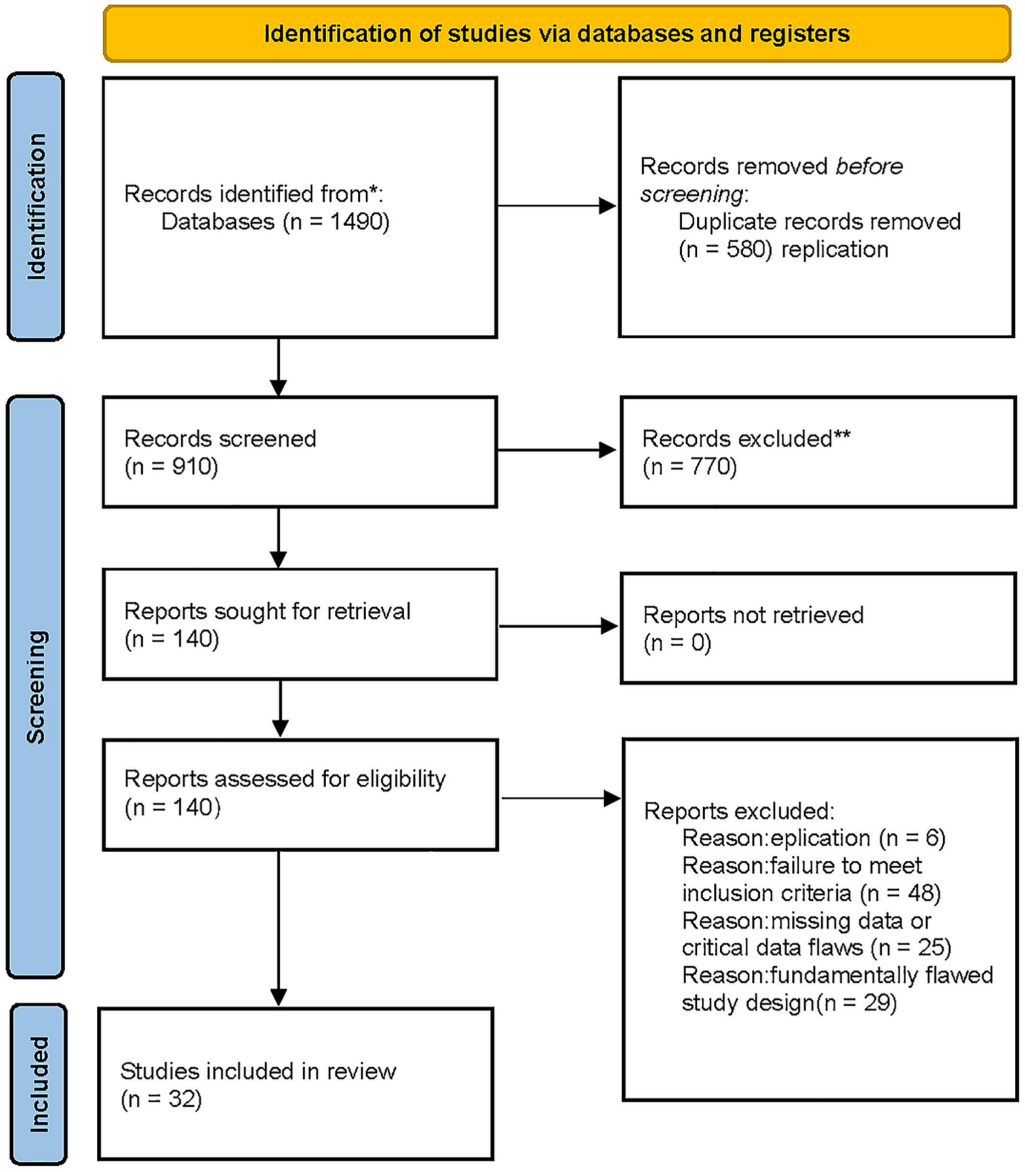
Literature screening process.

A total of 1,490 records were identified through database searches. After removal of 580 duplicates, 770 articles were excluded following preliminary screening of titles and abstracts. The remaining articles underwent full-text review, and studies were included or excluded strictly based on predefined inclusion and exclusion criteria. Ultimately, 32 studies were included in the analysis. The detailed screening process is illustrated in Figure 1.

### Characteristics and quality assessment of included studies

The 32 included studies (12–43) originated from one country (China), encompassing a total of 2,644 patients, of whom 52.87% were male and 47.13% female, with an age range of 60–86 years. The basic characteristics of the included studies are summarized in Table 1.

**Table 1:** Basic characteristics of included studies.

First author	Publication year	Region	Number of cases		Gender (male/female)	Age	Intervention (specific measures)		Treatment	Intervention time point	Outcome indicators	Type of surgery	Perioperative management protocol	Acupuncture parameters		
			Experimental group	Control group			Experimental group	Control group						Acupoint	Acupuncture time	
Xiaoqiu Gao	2012	China	60	60	52/68	71.65 ± 4.8373	Electroacupuncture	General Anesthesia	1d	Intraoperative	MMSE SCORE, NUMBER OF POCD OCCURRENCES	Non-cardiac surgery	Anesthetic Induction: Etomidate, Fentanyl, Cisatracurium; Anesthetic Maintenance: Sevoflurane Inhalation, Remifentanil	GV20, LI4, PC6, ST36	Initiated 30 min before induction until end of surgery	
Sunyan Lin	2013	China	38	37	48/27	68.5067 ± 3.5541	Electroacupuncture	General Anesthesia	1d	Intraoperative	MMSE SCORE, NUMBER OF POCD OCCURRENCES	Radical resection of colorectal cancer under general anesthesia	Anesthetic Induction: Midazolam, Propofol, Fentanyl, Vecuronium Bromide; Anesthetic Maintenance: Propofol, Remifentanil	GV20, PC6, ST36, SP6	Initiated 20 min before induction until end of surgery	
Sunyan Lin	2013	China	25	24	34/15	68.1573 ± 2.9428	Electroacupuncture	General Anesthesia	1d	Intraoperative	MMSE SCORE, NUMBER OF POCD OCCURRENCES	Open gastrointestinal tumor resection (colorectal resection)	Anesthetic Induction: Midazolam, Propofol, Fentanyl, Vecuronium; Anesthetic Maintenance: Propofol, Remifentanil	GV20, GV29, PC6	Initiated 30 min before induction until end of surgery	
Daiying Zhang	2014	China	60	60	61/59	73.325 ± 6.4737	Electroacupuncture	SoC	1d	Intraoperative	MMSE SCORE, NUMBER OF POCD OCCURRENCES	Major open abdominal surgery (radical colectomy, radical rectal resection, radical gastrectomy, subtotal gastrectomy)	Anesthetic Induction: Midazolam, Fentanyl, Propofol, Vecuronium Bromide; Anesthetic Maintenance: Propofol, Remifentanil, Cisatracurium Besylate	GV20, GV14, PC6	Initiated 30 min before induction until end of surgery	
Chenlin Zhang	2015	China	35	35	37/33	74.5 ± 4.0027	Electroacupuncture	General Anesthesia	8d	Postoperative	MMSE SCORE, NUMBER OF POCD OCCURRENCES	Radical resection of colorectal cancer under general anesthesia	Anesthetic Induction: Midazolam Propofol Fentanyl Vecuronium Bromide Anesthetic Maintenance: Propofol Remifentanil	GV20, PC6	Initiated 20 min before induction until end of surgery	
XIcheng Dong	2016	China	30	30	not mentioned	70 ± 4.601	Electroacupuncture	General Anesthesia	1d	Intraoperative	MMSE SCORE, NUMBER OF POCD OCCURRENCES	Rectal cancer surgery	Anesthetic Induction: Etomidate, Sufentanil, Rocuronium Bromide Anesthetic Maintenance: Propofol, Remifentanil, Rocuronium Bromide	GV20, PC6	Initiated 30 min before induction until end of surgery	
Zhi Liu	2017	China	49	49	48/50	73.935 ± 10.7366	Electroacupuncture	General Anesthesia	1d	Intraoperative	MMSE SCORE, NUMBER OF POCD OCCURRENCES	Geriatric tumor resection (gastric, hepatic, gallbladder cancers)	Anesthetic Induction: Midazolam, Penehyclidine Hydrochloride, Cisatracurium, Vecuronium Bromide Anesthetic Maintenance: Propofol, Remifentanil, Rocuronium Bromide	GV20, PC6, ST36	Initiated 30 min before induction until end of surgery	
Peina Zheng	2017	China	56	56	62/50	74.99 ± 4.2536	Electroacupuncture	General Anesthesia	1d	Intraoperative	MMSE SCORE, NUMBER OF POCD OCCURRENCES	Colorectal resection	/	GV20, PC6, ST36, SP6	Initiated before induction until end of surgery	
Gang Jin	2017	China	50	50	56/46	67.734 ± 2.5096	Electroacupuncture	General Anesthesia	1d	Intraoperative	MMSE SCORE, NUMBER OF POCD OCCURRENCES	Abdominal surgery	Anesthetic Induction: Midazolam, Propofol, Fentanyl, Vecuronium Bromide Anesthetic Maintenance: Propofol, Remifentanil	GV20, GV29, PC6	Initiated 30 min before induction until end of surgery	
Peirong Liu	2017	China	40	40	33/47	66.5 ± 6.4973	Electroacupuncture	General Anesthesia	7d	Perioperative	MMSE SCORE, NUMBER OF POCD OCCURRENCES	Hip arthroplasty	Anesthetic Induction: Fentanyl, Midazolam, Propofol, Cisatracurium Anesthetic Maintenance: Remifentanil, Propofol	LI4, LR3	30 min on non-surgical days. Initiated 30 min before induction until end of surgery on surgical days	
Kexue Zeng	2018	China	50	50	58/42	68.3 ± 1.2	Electroacupuncture	SoC	30d	Intraoperative	MMSE SCORE, NUMBER OF POCD OCCURRENCES	Lower abdominal or extremity surgery	Anesthetic Induction: Midazolam, Fentanyl, Vecuronium Bromide For Injection, Propofol Anesthetic Maintenance: Fentanyl, Vecuronium Bromide For Injection, Propofol, Isoflurane	GV20, GB20, BL23	20 min	
Feiyi Zhao	2018	China	30	30	26/34	65.965 ± 3.9724	Electroacupuncture	Placebo	5d	Preoperative	MMSE SCORE, NUMBER OF POCD OCCURRENCES	Knee arthroplasty	Anesthetic Induction: Fentanyl, Midazolam, Propofol, Cisatracurium Besilate Anesthetic Maintenance: Remifentanil, Propofol	GV20, GV24, EX-HN1, GB13, LI4, LR3	30 min	
Libing Zhang	2018	China	26	26	38/14	71 ± 7.9746	Xingnao Kaiqiao acnpnnctnr	SoC	7d	Postoperative	MMSE SCORE, NUMBER OF POCD OCCURRENCES	Hip arthroplasty under general anesthesia	Anesthetic Induction: Propofol, Remifentanil Hydrochloride, Sevoflurane, Midazolam, Vecuronium Bromide Anesthetic Maintenance: Propofol, Remifentanil Hydrochloride, Sevoflurane	GV26, PC6, SP6, LU5, HT1, BL40	/	
Xiaona Han	2018	China	45	45	50/40	68.25 ± 2.5246	Electroacupuncture	General Anesthesia	1d	Intraoperative	MMSEscore	Colorectal resection	Anesthetic Induction: Midazolam, Propofol, Fentanyl, Vecuronium Bromide Anesthetic Maintenance: Propofol, Remifentanil	GV20, SP6, ST36, PC6	Initiated 20 min before induction until end of surgery	
Haiyan Sun	2018	China	20	20/20	35/25	69.6 ± 2.3034	Electroacupuncture	General Anesthesia	1d	Intraoperative	MMSE SCORE, NUMBER OF POCD OCCURRENCES	Radical gastrectomy	Anesthetic Induction: Sufentanil, Cisatracurium, Etomidate Anesthetic Maintenance: Sevoflurane	PC6, LI4, ST36, ST37	Initiated 20 min before induction until end of surgery	
Qi Zhang	2018	China	45	45	48/42	72.5 ± 4.5302	Electroacupuncture	General Anesthesia	1d	Intraoperative	MMSEscore	Spinal surgery	Anesthetic Induction: Sufentanil, Midazolam, Etomidate, Cisatracurium Besilate Anesthetic Maintenance: Propofol, Remifentanil	GV20, GV14, ST36	Initiated 20 min before induction until end of surgery	
Ningke Wang	2018	China	48	48	58/38	68.55 ± 5.4748	Electroacupuncture	General Anesthesia	1d	Intraoperative	MMSEscore	Laparoscopic-assisted proximal subtotal gastrectomy	Anesthetic Induction: Midazolam, Fentanyl, Vecuronium Bromide, Propofol Anesthetic Maintenance: Fentanyl, Propofol, Vecuronium Bromide	PC6, LI4, ST36, ST37	Initiated 15–20 min before induction until end of surgery	
Qingguo Xu	2019	China	50	50	61/39	77.7 ± 5.9015	Electroacupuncture	General Anesthesia	1d	Intraoperative	MMSE SCORE, NUMBER OF POCD OCCURRENCES	Total hip arthroplasty	Anesthetic Induction: Midazolam, Sufentanil, Propofol, Rocuronium Bromide Anesthetic Maintenance: Remifentanil, Propofol	LI4, LR3	Initiated 30 min before induction until end of surgery	
Suping Yuan	2019	China	50	50	53/47	60–81	Electroacupuncture	General Anesthesia	1d	Intraoperative	MMSE SCORE, NUMBER OF POCD OCCURRENCES	Laparoscopic cholecystectomy	Anesthetic Induction: Midazolam, Sufentanil, Propofol, Rocuronium Bromide Anesthetic Maintenance: Remifentanil, Propofol	GV20, PC6	20 min	
Hongnan Wang	2019	China	42	42	49/35	63 ~ 79	Electroacupuncture	General Anesthesia	1d	Intraoperative	MMSEscore	Orthopedic surgery	Anesthetic Induction: Midazolam, Propofol, Fentanyl, Vecuronium Anesthetic Maintenance: Midazolam, Propofol, Fentanyl, Vecuronium	GV20, ST36, PC6	Initiated 30 min before induction until end of surgery	
Na Zhao	2021	China	30	30	33/27	71.58 ± 4.6161	Electroacupuncture	SoC	7d	Postoperative	MMSE SCORE, NUMBER OF POCD OCCURRENCES	/	Anesthetic Induction: Midazolam, Cisatracurium, Fentanyl, Propofol Anesthetic Maintenance: Sevoflurane, Propofol, Remifentanil, Cisatracurium	GV20, HT7, PC6, LI4	20 min	
Yanhua Tang	2022	China	30	30	32/28	69.155 ± 3.5597	Electroacupuncture	General Anesthesia	9d	Intraoperative	MMSE SCORE, NUMBER OF POCD OCCURRENCES	Hip arthroplasty, tibial fracture fixation, femoral fracture fixation	/	KI1, HT7, GV20	30 min	
Ronghua Li	2022	China	40	40	18/62	73 ± 4.1	Thumbtack needle	Placebo	3D	Preoperative	MMSE SCORE, NUMBER OF POCD OCCURRENCES	Hip fracture surgery	Anesthetic Induction: Propofol, Citric Acidsufentanil, Cisatracurium, Midazolam; Anesthetic Maintenance: Propofol, Remifentanil, Dexmedetomidine, Sevoflurane	GV20, HT7, LI4, PC6, ST36	5 times daily, 2 min each	
Meihuang Cai	2022	China	58	59	59/58	68.5043 ± 3.5516	Scalp Acupuncture	SoC	3d	Intraoperative	MMSEscore	Intertrochanteric femoral fracture surgery	/	(GV24.5 → GV20), (GV20 → GV21)	Sustained stimulation throughout the surgical procedure	
Yongda Luo	2022	China	60	60	45/75	71.525 ± 5.0811	Electroacupuncture	General Anesthesia	3d	Postoperative	MMSE SCORE, NUMBER OF POCD OCCURRENCES	/	/	GV20, PC6, ST36	30 min	
Yiqin Wan	2023	China	30	30	39/21	73 ± 8.5022	Electroacupuncture	General Anesthesia	1d	Preoperative	MMSE SCORE, NUMBER OF POCD OCCURRENCES	Flexible ureteroscopic holmium laser lithotripsy	Anesthetic Induction: Midazolam, Sufentanil, Propofol, Cisatracurium Besylate; Anesthetic Maintenance: Propofol, Remifentanil, with supplemental Cisatracurium Besylate	ST36, PC6	30 min	
Jie Zheng	2023	China	30	30	38/22	70.55 ± 1.4394	Electroacupuncture	SoC	6d	Perioperative	MMSE SCORE, NUMBER OF POCD OCCURRENCES	Hip arthroplasty under general anesthesia	Anesthetic Induction: Fentanyl, Midazolam, Propofol, Cisatracurium Anesthetic Maintenance: Propofol, Remifentanil, with supplemental Cisatracurium	LI4, LR3	30 min	
Linyun Zhuang	2023	China	32	29	Not mentioned	80.7982 ± 5.311	Acupunture	General Anesthesia	1d	Preoperative	MMSE score	Hip fracture surgery	Anesthetic Induction: Midazolam, Citric Acidfentanyl, Cisatracurium Besylate, Propofol Anesthetic Maintenance: Remifentanil, Propofol	HT5, PC6, ST40	Twice daily	
Linyuan Song	2023	China	20	20	23/17	63.685 ± 2.8211	Xingnao Kaiqiao acnpnnctnr	SoC	5d	Postoperative	MMSEscore	/	/	PC6, GV26, SP6 BL40, HT1, LU5	/	
Jian Xie	2023	China	45	45	54/36	66.99 ± 6.0331	Electroacupuncture	General Anesthesia	1d	Intraoperative	MMSE SCORE, NUMBER OF POCD OCCURRENCES	Radical rectal cancer resection	Anesthetic Induction: Propofol, Sufentanil, Vecuronium Bromide Anesthetic Maintenance: Propofol, Remifentanil	ST36, PC6, SP6, GV20	Initiated 30 min before induction until end of surgery	
Yong Liu	2023	China	60	60	56/64	72.31 ± 5.7236	Electroacupuncture	General Anesthesia	1d	Intraoperative	MMSE score	Hip arthroplasty	Anesthetic Induction: Midazolam, Citric Acidfentanyl, Propofol, Vecuronium Bromide Anesthetic Maintenance: Remifentanil, Vecuronium Bromide, Propofol	GB41, GB31, Dai Mai, Ah Shi Point	Throughout the surgical procedure	
Meini Wang	2024	China	30	30	31/29	74.02 ± 5.4178	Ear acupuncture	SoC	7d	Perioperative	MMSE score	Hip arthroplasty	/	MA-IC, MA-TF1, MA-AT, MA-LO, MA-AH|		20–30 min

SoC, Standard of Care; MMSE, Mini-Mental State Examination.

**Figure 2:**
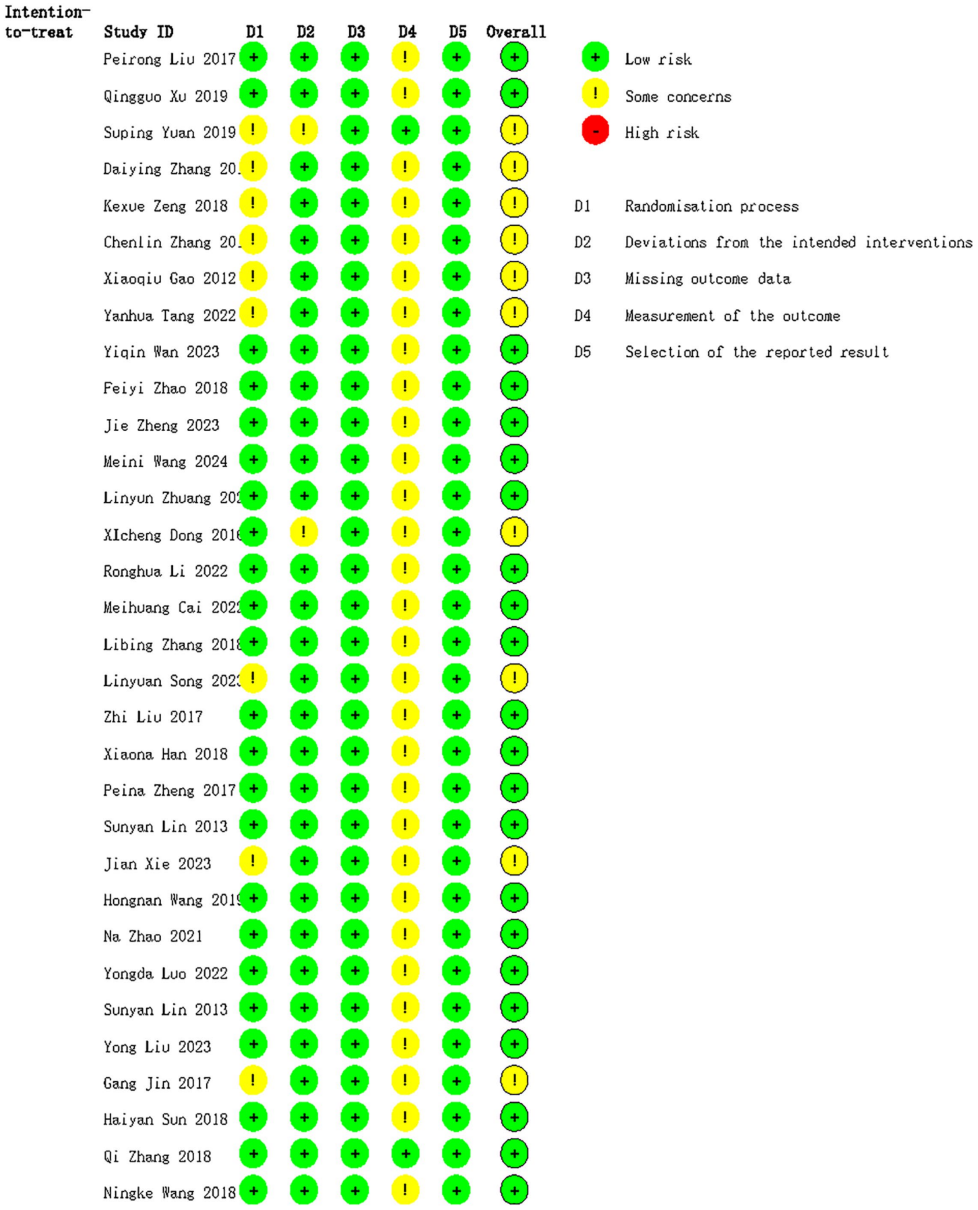
Methodological quality assessment of included studies.

Risk of bias assessment indicated that the majority of studies were at low risk. Ten studies (14–19, 25, 29, 34, 39) were judged to have moderate risk, with eight studies (15–19, 29, 34, 39) having moderate risk primarily due to unreported allocation concealment and unclear blinding of outcome assessors, which may have affected outcome measurement. The first author (Liang) and second author (Li) discussed these cases but could not reach a consensus; following consultation with the third author (Zhang), these were classified as moderate risk. One study (14) was assigned moderate risk because of unspecified allocation concealment and lack of blinding for trial implementers, with unanimous agreement among the authors. Another study (25) was also classified as moderate risk due to absence of information on blinding of implementers and outcome assessors; after discussion among the authors, it was rated as moderate risk. The methodological quality assessment for each trial is presented in Figure 2.

### Network meta-analysis results

#### Network geometry

**Figure 3:**
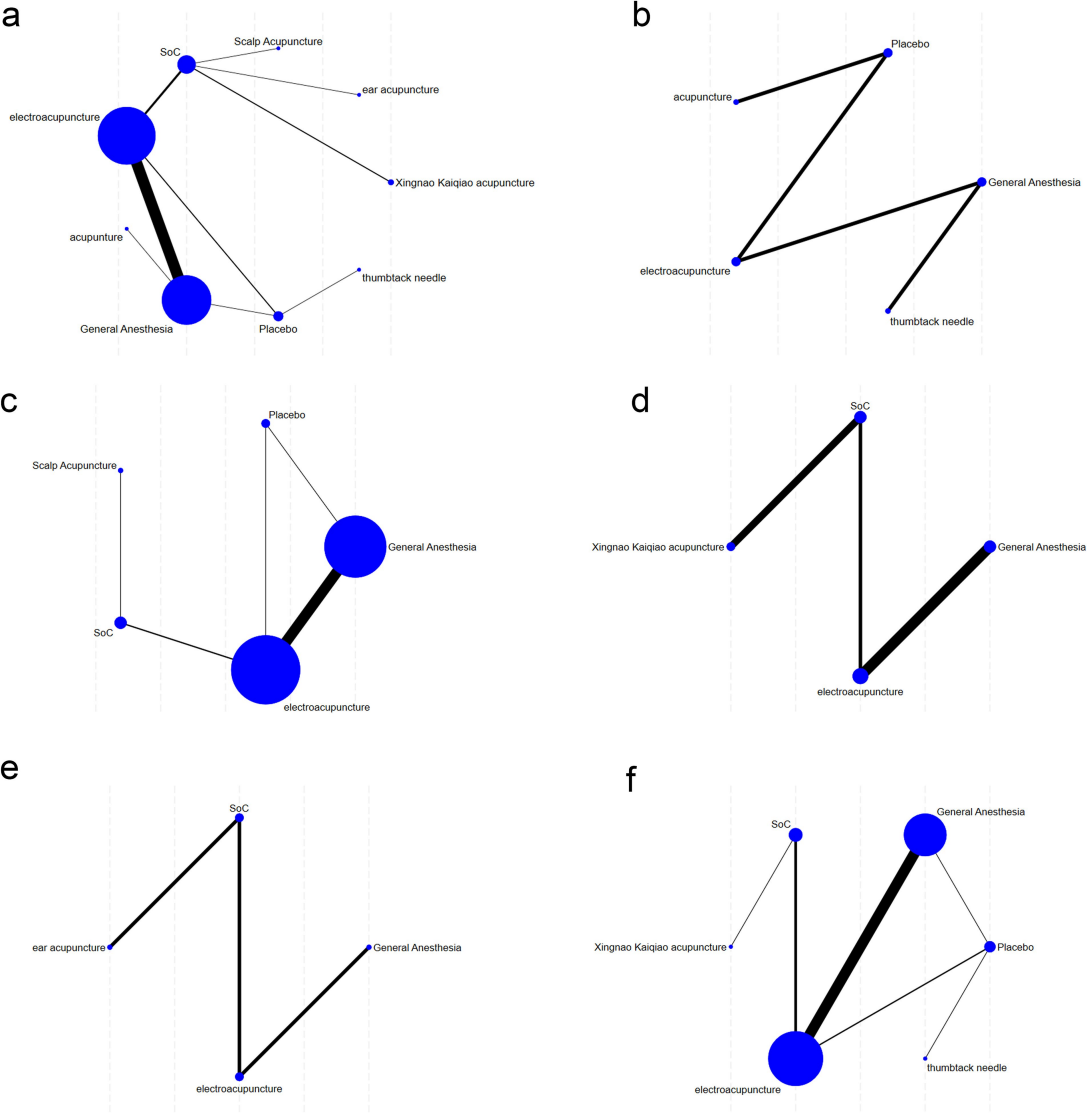
Network relationship diagram. **(a)** MMSE, **(b)** preoperative intervention subgroup, **(c)** intraoperative intervention subgroup, **(d)** postoperative intervention subgroup, **(e)** perioperative intervention subgroup, and **(f)** number of POCD events.

In the network plots, each node represents an intervention, with node size proportional to the number of included studies investigating that intervention. Edges connecting nodes indicate direct comparisons between interventions; edge thickness reflects the number of studies for each comparison, with thicker lines representing more evidence (see Figure 3). The posterior residual deviance (PRSF) for all analyses was equal to 1, indicating model convergence. Node-splitting analyses were conducted for all closed loops, showing *p*-values > 0.05, thus demonstrating consistency between direct and indirect evidence.

### Summary of outcome indicators

#### MMSE

**Figure 4:**
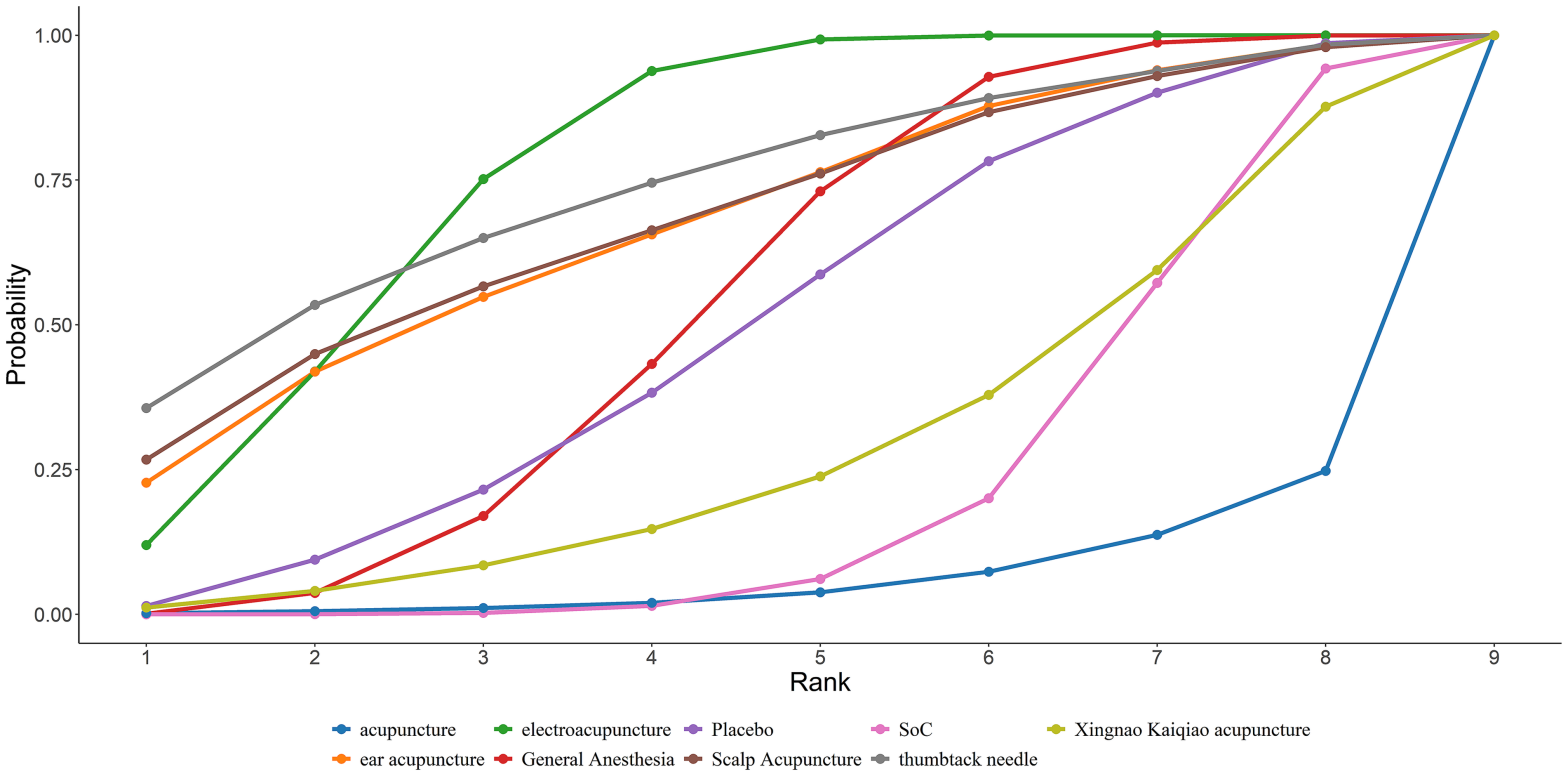
SUCRA curve of the effects of different acupuncture modalities on MMSE in elderly patients undergoing general anesthesia surgery.

A total of 32 studies reported MMSE scores. Under the random-effects model within the network meta-analysis (NMA) framework, results indicated that: General anesthesia was associated with a significant decline in postoperative cognitive function MMSE scores compared with electroacupuncture (General Anesthesia vs. Electroacupuncture: MD = −0.70; 95% CrI: −1.31 to −0.09). Similarly, Standard of Care (SoC) showed a significant decrease in MMSE scores compared to electroacupuncture (SoC vs. Electroacupuncture: MD = −1.98; 95% CrI: −3.38 to −0.57). Conversely, general anesthesia yielded significantly higher MMSE scores than acupuncture (General Anesthesia vs. Acupuncture: MD = 2.96; 95% CrI: 0.10–5.82). Electroacupuncture was also superior to acupuncture (Electroacupuncture vs. Acupuncture: MD = 3.66; 95% CrI: 0.73–6.58). Detailed pairwise comparisons are provided in Table 2. The SUCRA rankings indicated the best efficacy of electroacupuncture in reducing postoperative MMSE decline among elderly patients undergoing general anesthesia: Electroacupuncture (77.93%) > Thumbtack Needle (73.89%) > Scalp Acupuncture (68.58%) (see Figure 4). MMSE overall ranking is shown in Table 3.

**Table 2:** Pairwise comparisons for the effects of various acupuncture modalities on MMSE scores in elderly patients undergoing general anesthesia surgery.

General anesthesia								
1.28 (−0.26, 2.81)	SoC							
**−0.7 (−1.31, −0.09)**	**−1.98 (−3.38, −0.57)**	Electroacupuncture						
0.18 (−1.71, 2.08)	−1.1 (−3.41, 1.23)	0.88 (−0.97, 2.73)	Placebo					
−0.59 (−3.77, 2.59)	−1.87 (−4.64, 0.91)	0.11 (−3, 3.22)	−0.76 (−4.4, 2.83)	Ear acupuncture				
−0.95 (−4.3, 2.4)	−2.23 (−5.84, 1.38)	−0.25 (−3.58, 3.08)	−1.13 (−3.91, 1.63)	−0.37 (−4.91, 4.21)	Thumbtack needle			
−0.66 (−4.07, 2.75)	−1.94 (−4.98, 1.1)	0.04 (−3.31, 3.4)	−0.84 (−4.68, 2.99)	−0.07 (−4.19, 4.04)	0.29 (−4.46, 5.02)	Scalp acupuncture		
**2.96 (0.1, 5.82)**	1.68 (−1.56, 4.92)	**3.66 (0.73, 6.58)**	2.77 (−0.65, 6.19)	3.54 (−0.72, 7.81)	3.9 (−0.49, 8.31)	3.62 (−0.82, 8.06)	Acupuncture	
1.33 (−1.21, 3.91)	0.05 (−1.98, 2.12)	2.03 (−0.43, 4.53)	1.15 (−1.94, 4.26)	1.92 (−1.51, 5.38)	2.28 (−1.85, 6.46)	1.99 (−1.66, 5.66)	−1.63 (−5.44, 2.23)	Xingnao Kaiqiao acupuncture

Bolded values signify that the difference in MMSE changes between the column intervention and the row intervention is statistically significant.

**Table 3:** Summary of MMSE and POCD rankings.

	1	2	3	4	5	6	7	8	9
MMSE	Electroacupuncture (77.93%)	Thumbtack needle (73.89%)	Scalp Acupuncture (68.58%)	Ear acupuncture (67.70%)	General Anesthesia (53.59%)	Placebo (49.55%)	Xingnao Kaiqiao acupuncture (29.67%)	SoC (22.42%)	Acupuncture (6.7%)
MMSE (Preoperative)	Electroacupuncture (73.01%)	General Anesthesia (67.42%)	Thumbtack needle (61.77%)	Placebo (33.32%)	Acupuncture (14.47%)				
MMSE (Intraoperative)	Electroacupuncture (76.78%)	Scalp Acupuncture (65.03%)	Placebo (55.35%)	General Anesthesia (40.74%)	SoC (12.10%)				
MMSE (Postoperative)	Electroacupuncture (82.46%)	General Anesthesia (71.68%)	Xingnao Kaiqiao acupuncture (25.92%)	SoC (19.94%)					
MMSE (Perioperative)	Ear acupuncture (84.64%)	Electroacupuncture (66.32%)	SoC (25.24%)	General Anesthesia (23.79%)					
POCD	Xingnao Kaiqiao acupuncture (86.56%)	Thumbtack needle (80.16%)	Electroacupuncture (58.78%)	SoC (38.44%)	Placebo (27.78%)	General Anesthesia (8.26%)			

### Subgroup analyses by intervention timing and modality

#### Preoperative interventions

**Figure 5:**
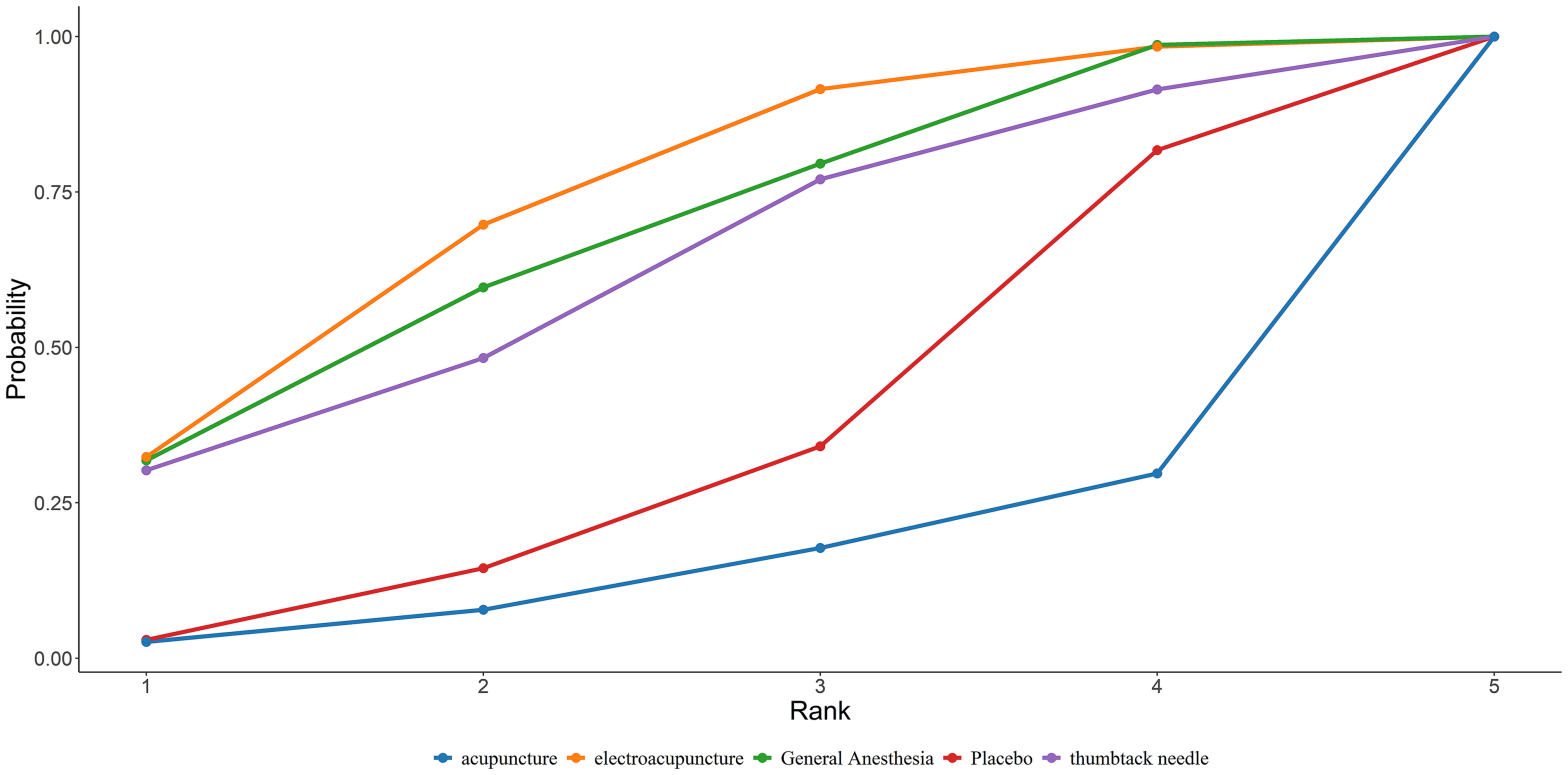
SUCRA curve of the effects of different preoperative acupuncture on MMSE in elderly patients undergoing general anesthesia surgery.

Network meta-analysis of four studies with preoperative interventions is summarized in Table 4. SUCRA rankings were: Electroacupuncture (73.01%) > General Anesthesia (67.42%) > Thumbtack Needle (61.77%). Electroacupuncture demonstrated the most favorable effect in reducing postoperative MMSE decline preoperatively (Figure 5). The preoperative MMSE ranking is presented in Table 3.

**Table 4:** Pairwise comparisons for the effects of various acupuncture modalities during surgery on MMSE scores in elderly patients undergoing general anesthesia.

General anesthesia				
−0.19 (−4.05, 3.69)	Electroacupuncture			
1.39 (−4.06, 6.86)	1.58 (−2.28, 5.42)	Placebo		
0.26 (−6.31, 6.9)	0.45 (−4.91, 5.84)	−1.13 (−4.86, 2.64)	Thumbtack needle	
2.95 (−0.91, 6.84)	3.14 (−2.34, 8.59)	1.57 (−5.15, 8.26)	2.69 (−5, 10.35)	Acupuncture

Bold values signify that the difference in MMSE changes between the column intervention and the row intervention is statistically significant.

#### Intraoperative

**Figure 6:**
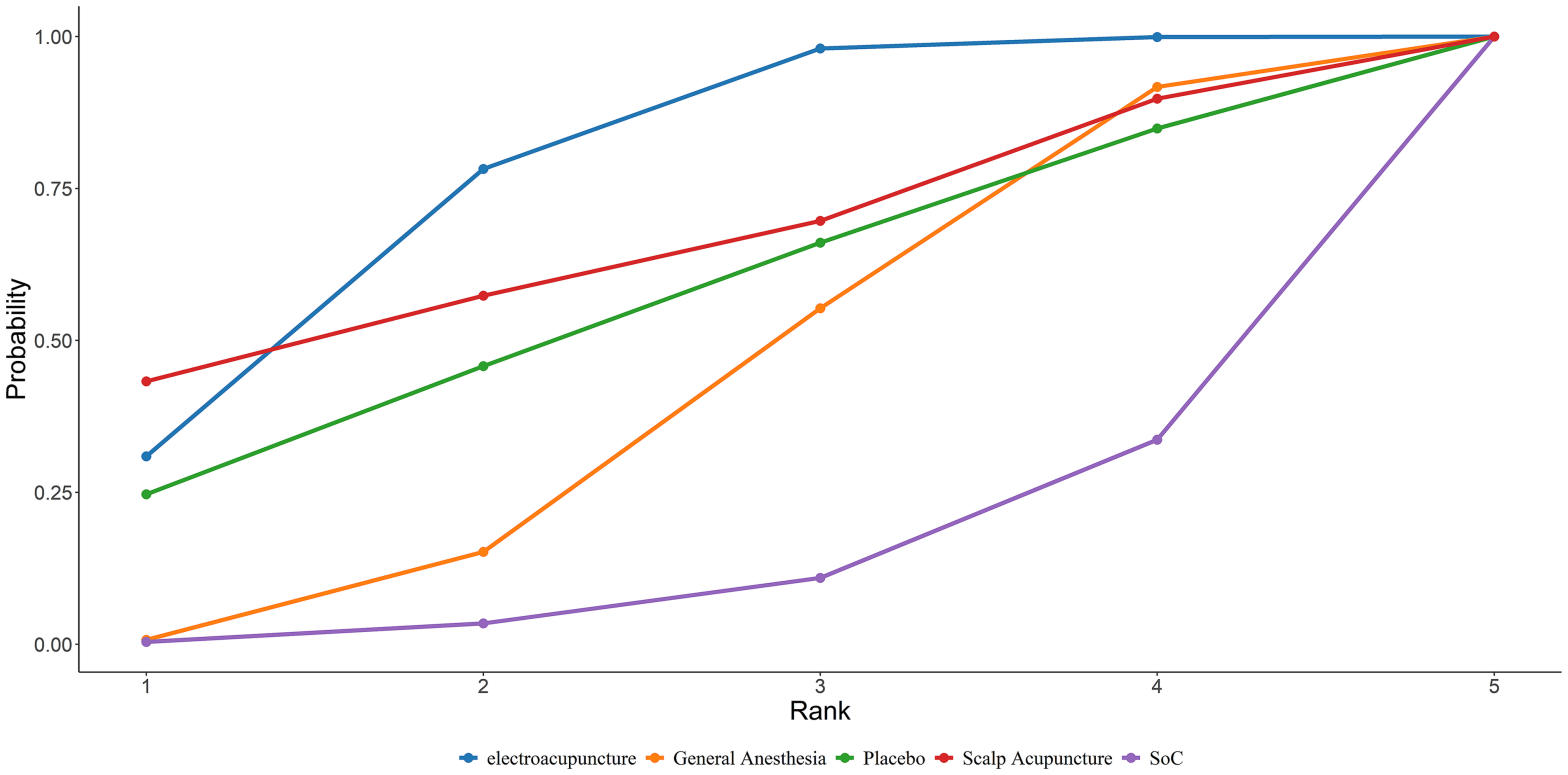
Area under the SUCRA curve of the effects of different acupuncture modalities on MMSE in elderly patients undergoing general anesthesia surgery during operation.

Nineteen studies assessing intraoperative interventions were analyzed (Table 5). SUCRA rankings were: Electroacupuncture (76.78%) > Scalp Acupuncture (65.03%) > Placebo (55.35%). Electroacupuncture was most effective in attenuating intraoperative cognitive decline (Figure 6). The intraoperative MMSE ranking is summarized in Table 3.

**Table 5:** Pairwise comparisons for the effects of various acupuncture modalities during surgery on MMSE scores in elderly patients under general anesthesia.

General anesthesia				
1.18 (−1.12, 3.47)	SoC			
−0.77 (−1.55, 0.01)	−1.95 (−4.11, 0.21)	Electroacupuncture		
−0.34 (−3.07, 2.4)	−1.52 (−5, 1.96)	0.43 (−2.3, 3.16)	Placebo	
−0.77 (−4.8, 3.26)	−1.94 (−5.25, 1.36)	0.01 (−3.95, 3.95)	−0.42 (−5.24, 4.37)	Scalp acupuncture

Bold values signify that the difference in MMSE changes between the column intervention and the row intervention is statistically significant.

#### Postoperative

Six studies on postoperative interventions were included (Table 6). SUCRA rankings showed: Electroacupuncture (82.46%) > General Anesthesia (71.68%) > Xingnao Kaiqiao Acupuncture (25.92%). Electroacupuncture exhibited the greatest benefit postoperatively (Supplementary Figure S1). The postoperative MMSE ranking is displayed in Table 3.

**Table 6:** Pairwise comparisons for the effects of various postoperative acupuncture modalities on MMSE scores in elderly patients undergoing general anesthesia surgery.

General anesthesia			
2.62 (−1.72, 6.99)	SoC		
−0.27 (−2.43, 1.91)	−2.88 (−6.65, 0.88)	Electroacupuncture	
2.44 (−2.5, 7.88)	−0.16 (−2.75, 2.87)	2.71 (−1.76, 7.65)	Xingnao Kaiqiao acupuncture

Bold values signify that the difference in MMSE changes between the column intervention and the row intervention is statistically significant.

#### Perioperative interventions

Seven studies involving perioperative interventions were analyzed (Table 7). SUCRA rankings indicated: Ear Acupuncture (84.64%) > Electroacupuncture (66.32%) > SoC (25.24%). Ear acupuncture had the best effect in reducing postoperative MMSE decline during the perioperative period (Supplementary Figure S2). The perioperative MMSE ranking is also provided in Table 3.

**Table 7:** Pairwise comparisons for the effects of various acupuncture modalities during surgery on MMSE scores in elderly patients undergoing general anesthesia.

General anesthesia			
−0.13 (−3.69, 3.41)	SoC		
−1.2 (−3.65, 1.25)	−1.07 (−3.64, 1.5)	Electroacupuncture	
−2 (−6.29, 2.28)	−1.87 (−4.29, 0.54)	−0.8 (−4.33, 2.71)	Ear acupuncture

Bold values indicate that the difference in MMSE changes between the column intervention and the row intervention is statistically significant.

#### Incidence of POCD

**Figure 7:**
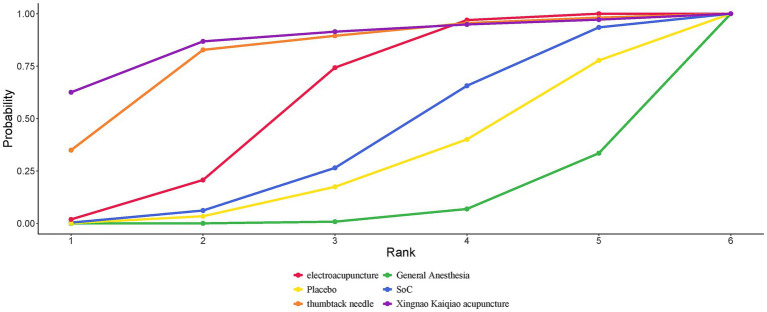
SUCRA curves for the incidence of POCD in elderly patients undergoing general anesthesia treated with different acupuncture modalities.

A total of 23 studies reported the incidence of POCD. Based on the Bayesian network meta-analysis (NMA) within a random-effects framework, the results demonstrated that the incidence of POCD postoperatively in elderly patients receiving general anesthesia was significantly higher compared to those treated with electroacupuncture (General Anesthesia vs. Electroacupuncture: RR = 2.15, 95% CrI = 1.52–3.18; see Table 8). The SUCRA ranking probabilities indicated that Xingnao Kaiqiao acupuncture (86.56%) ranked highest, followed by thumbtack needle (80.16%), and electroacupuncture (58.78%). Thus, Xingnao Kaiqiao acupuncture was associated with the lowest postoperative POCD incidence in elderly patients undergoing general anesthesia (Figure 7). The comparative ranking of POCD outcomes is summarized in Table 3.

**Table 8:** Pairwise comparison of postoperative POCD incidence among elderly patients undergoing general anesthesia with different acupuncture interventions.

General anesthesia					
1.62 (0.76, 3.65)	SoC				
1.37 (0.52, 3.78)	0.84 (0.26, 2.7)	Placebo			
**2.15 (1.52, 3.18)**	1.33 (0.66, 2.62)	1.57 (0.61, 4.05)	Electroacupuncture		
5.05 (0.87, 37.04)	3.11 (0.47, 24.54)	3.65 (0.86, 20.54)	2.34 (0.41, 16.67)	Thumbtack needle	
8.6 (0.87, 265.02)	5.21 (0.61, 148.6)	6.31 (0.54, 214.83)	3.97 (0.41, 120.28)	1.74 (0.08, 74.55)	Xingnao Kaiqiao acupuncture

Values in bold denote statistically significant differences in POCD incidence between the column and row interventions.

#### Publication bias

**Figure 8:**
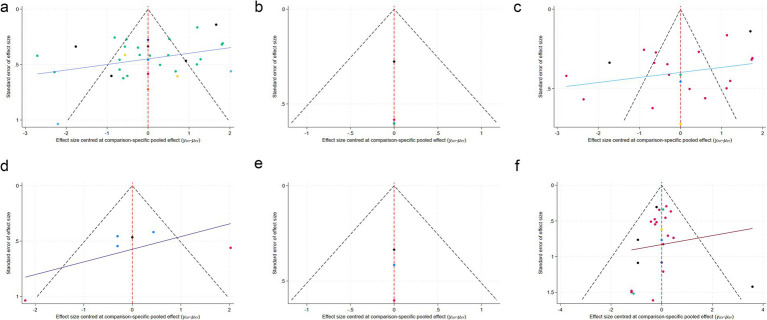
Funnel plots for publication bias assessment. **(a)** MMSE; **(b)** preoperative intervention subgroup; **(c)** intraoperative intervention subgroup; **(d)** postoperative intervention subgroup; **(e)** perioperative intervention subgroup; **(f)** POCD incidence.

Publication bias was evaluated using contour-enhanced funnel plots, Egger’s test, and Begg’s test. For MMSE, the funnel plot appeared symmetrical (Figure 8); however, Egger’s test yielded statistical significance (*p* = 0.001), suggesting potential publication bias, whereas Begg’s test did not (*p* = 0.236). For POCD, the funnel plot was symmetric (Figure 8), and both Egger’s test (*p* = 0.64) and Begg’s test (*p* = 0.493) indicated no publication bias.

## Discussion

This meta-analysis incorporated 32 trials encompassing 2,644 elderly patients undergoing general anesthesia. Using a Bayesian network meta-analytic approach, we compared the efficacy of Electroacupuncture, Scalp Acupuncture, Ear Acupuncture, Acupuncture, Thumbtack Needle, Xingnao Kaiqiao Acupuncture, General Anesthesia, Standard of Care (SoC), and Placebo in mitigating postoperative declines in MMSE scores. At present, there is no universally accepted diagnostic criterion for POCD. In the included studies, POCD diagnosis relied on changes in MMSE scores from pre- to postoperative assessments. Although the Montreal Cognitive Assessment (MoCA) demonstrates higher sensitivity, evidence remains limited, and its diagnostic utility for POCD requires further validation. Consequently, MMSE was adopted as the primary outcome measure in this study. The objective was to provide evidence-based guidance for selecting acupuncture interventions in elderly patients undergoing general anesthesia. Key findings are summarized as follows: All analyses indicated that Electroacupuncture ranked highest in overall efficacy, followed by Thumbtack Needle and Scalp Acupuncture. Subgroup analyses based on intervention timing revealed that: Preoperative stage: Electroacupuncture was significantly superior to General Anesthesia and Thumbtack Needle; Intraoperative stage: Electroacupuncture demonstrated the best efficacy, outperforming Scalp Acupuncture and Placebo; Postoperative stage: Electroacupuncture remained the most effective, exceeding General Anesthesia and Xingnao Kaiqiao Acupuncture; Perioperative stage: Ear Acupuncture showed significant advantage, superior to Electroacupuncture and SoC.

For the secondary outcome of postoperative POCD incidence, 23 trials involving 1,886 elderly patients were included. The Bayesian network meta-analysis compared Electroacupuncture, Thumbtack Needle, Xingnao Kaiqiao Acupuncture, General Anesthesia, SoC, and Placebo. Results indicated that Xingnao Kaiqiao Acupuncture was associated with the lowest incidence of POCD.

Electroacupuncture, which integrates traditional acupuncture with modern electrical stimulation by delivering microcurrents at specific frequencies and intensities to acupoints, was investigated in 27 studies included herein. It ranked first in efficacy across all interventions and in subgroup analyses of preoperative, intraoperative, and postoperative periods, ranking second only in the perioperative subgroup. Notably, only two studies in the perioperative subgroup employed electroacupuncture interventions spanning pre-, intra-, and postoperative phases, while the remaining studies applied electroacupuncture at a single time point. These findings suggest that the timing and frequency of electroacupuncture may be critical determinants of its effectiveness. Our analysis confirms the significant efficacy of electroacupuncture in preventing postoperative cognitive dysfunction in elderly patients undergoing general anesthesia, supporting its clinical application for this indication. However, methodological quality varied across included electroacupuncture studies, with common deficiencies such as inadequate allocation concealment and lack of blinding, potentially introducing selection and performance biases. While current evidence is promising, further high-quality randomized controlled trials are needed to strengthen the evidence base.

The neuroprotective mechanisms of electroacupuncture likely involve inhibition of inflammation, reduction of oxidative stress, neuronal protection, and synaptic remodeling, thereby modulating POCD pathophysiology (44). In a murine femoral fracture surgical model, electroacupuncture significantly decreased peripheral and central inflammatory markers and improved cognitive function (45, 46). In a rat model of abdominal surgery, electroacupuncture pretreatment markedly increased hippocampal neuronal counts in aged rats and protected mitochondrial structure and function by reducing intracellular calcium levels, thus inhibiting neuronal apoptosis (47). Additionally, electroacupuncture may indirectly regulate central nervous system inflammation by modulating gut microbiota composition and their metabolites (e.g., short-chain fatty acids), as well as epigenetically modulating cognition-related gene expression through DNA methylation or histone modifications (47).

Thumbtack Needle is a traditional Chinese medicine external treatment tool resembling a miniature thumbtack, composed of a 1–2 mm short needle affixed to an adhesive patch base. It is secured to the skin surface at acupoints via medical tape to provide continuous mild stimulation. It is commonly used for alleviating chronic pain, regulating insomnia, anxiety, and related conditions. Thumbtack needle therapy modulates pain transmission pathways and inhibits the release of inflammatory mediators such as C-reactive protein (CRP) and prostaglandin E2 (PGE2) through sustained acupoint stimulation (48). This modality has demonstrated comparable efficacy to conventional analgesia in postoperative pain management following cesarean section (49), total knee arthroplasty (48), and laparoscopic hysterectomy (50). The widely accepted “microglia–neuroinflammation–cognitive dysfunction” pathway suggests that surgical stimuli activate central nervous system microglia, bone marrow-derived macrophages (BMDMs), mast cells, and T lymphocytes, resulting in the release of proinflammatory cytokines concentrated in specific brain regions. This cascade exacerbates postoperative neuroinflammation and subsequently induces cognitive impairment (51). Thumbtack needle therapy can alleviate postoperative pain, especially inflammatory pain, thereby reducing postoperative complications and intraoperative anesthetic requirements, and improving postoperative recovery quality. Effective analgesia may attenuate inflammation, thus mitigating pain-associated postoperative cognitive dysfunction (POCD). However, to date, only one study has evaluated the effect of thumbtack needle on cognitive impairment in elderly patients undergoing general anesthesia.

Scalp Acupuncture (Head Needle) is a specialized form of acupuncture that targets specific stimulation zones on the scalp, aiming to modulate central nervous system function by stimulating cerebral cortex projection areas on the scalp surface. It has been shown to improve memory function, with related studies elucidating its underlying biological mechanisms. Scalp acupuncture targets the left angular gyrus (Brodmann area 39) and fusiform gyrus (Brodmann area 37), modulating brain regions associated with semantic processing and memory retrieval, based on projection correlation analysis from neuroimaging databases (52). In ischemic cerebrovascular disease models, scalp acupuncture promotes microglial polarization from the pro-inflammatory M1 phenotype to the anti-inflammatory M2 phenotype, thereby alleviating neuroinflammatory injury (53). Additionally, it downregulates interferon-gamma (IFN-γ) expression, inhibits the JAK/STAT1 signaling pathway, and regulates IL-12-mediated inflammatory cascades (54). These mechanisms collectively contribute to improved memory function in ischemic cerebrovascular conditions. Scalp acupuncture may exert protective effects against POCD by suppressing neuroinflammatory pathways. Nonetheless, only one study to date has explored the effect of scalp acupuncture on cognitive dysfunction in elderly patients undergoing general anesthesia. Hence, more high-quality, large-sample RCTs with rigorous design are necessary to further investigate its clinical efficacy in this context.

Our analysis revealed that thumbtack needle acupuncture and Xingnao Kaiqiao acupuncture substantially reduced the incidence of POCD. Nevertheless, their rankings in MMSE outcomes were inconsistent. Specifically, MMSE analysis incorporated 32 trials with 2,644 patients, whereas POCD incidence analysis included only 23 trials with 1,886 patients. The smaller sample size for POCD may have undermined statistical power and obscured the true therapeutic effects of thumbtack needle and Xingnao Kaiqiao acupuncture. Variability in intervention timing and duration across acupuncture modalities may also account for divergent findings between MMSE and POCD outcomes.

This study represents the first network meta-analysis (NMA) evaluating the efficacy of various acupuncture modalities in mitigating postoperative MMSE score decline among elderly patients undergoing general anesthesia, thereby providing clinical reference for the treatment of postoperative cognitive dysfunction. Moreover, we analyzed the ranking of acupuncture efficacy according to different intervention timings. However, several limitations should be acknowledged. Some included studies carried a moderate risk of bias (due to inadequate allocation concealment and lack of blinding), which may weaken the validity of the results. The absence of allocation concealment increases the risk of selection bias, potentially leading to overestimation of treatment effects; the lack of blinding may compromise the objectivity and accuracy of subjective outcome measures. The study population consisted exclusively of Chinese patients. While such homogeneity enhanced statistical efficiency, it markedly restricted external validity. Differences in genetic background, perioperative medication practices, depth-of-anesthesia monitoring, and postoperative rehabilitation protocols across countries may influence the effectiveness of acupuncture for POCD. Direct head-to-head comparisons of different acupuncture modalities were unavailable, precluding definitive comparative conclusions. Some studies were limited by small sample sizes, thereby increasing the likelihood of bias. Follow-up time points for outcome assessment were inconsistent across studies. Reliance on a single outcome measure introduces subjectivity and restricts sensitivity, especially for detecting early or subtle cognitive decline. Although comparing pre- and postoperative MMSE scores permits detection of substantial cognitive deterioration, MMSE lacks sensitivity for early or subtle impairments, particularly in executive function, processing speed, and working memory. Consequently, mild or subclinical deficits may have been underestimated or overlooked. This study focused exclusively on the effect of acupuncture modality on POCD without investigating the influence of acupoint selection, which is also a critical determinant. Furthermore, POCD occurrence is closely associated with patient-related factors, surgical variables, anesthetic agents, and anesthesia-related risk factors. Future research should conduct multicenter RCTs directly comparing different acupuncture therapies while integrating multidimensional data such as patient baseline characteristics and perioperative anesthetic parameters to establish risk stratification models for POCD. Additionally, future studies should incorporate neuroinflammatory biomarkers alongside MMSE assessments and extend follow-up to at least 3 months postoperatively to evaluate long-term outcomes. Emphasis should be placed on elucidating (1) the specific neuroprotective effects of different acupuncture methods and acupoint combinations during surgery, and (2) how these modalities might synergistically enhance therapeutic efficacy. Such evidence will enable clinicians to select the optimal acupuncture strategy tailored to individual patients to prevent POCD.

## Conclusion

In conclusion, this study indicated that electroacupuncture provided the most robust protection against postoperative MMSE decline in elderly Chinese patients undergoing general anesthesia. Thumbtack needle and scalp acupuncture demonstrated relatively favorable effects as well. Electroacupuncture was consistently the most effective intervention across preoperative, intraoperative, and postoperative phases. Nevertheless, given the methodological limitations, future research should prioritize large-scale, rigorously designed, multicenter randomized controlled trials across diverse populations and cultural contexts to validate the generalizability and safety of acupuncture interventions. Moreover, adoption of more sensitive cognitive assessment instruments, such as the MoCA, should be pursued to enhance diagnostic precision in POCD. Future research should further clarify the impacts of electroacupuncture, thumbtack needle, scalp acupuncture, and intervention timing on postoperative cognitive dysfunction in elderly surgical patients under general anesthesia.

## Data Availability

The original contributions presented in the study are included in the article/Supplementary material, further inquiries can be directed to the corresponding author.
