# Supplementary material for: The effects of different acupuncture modalities on postoperative cognitive function in elderly Chinese patients undergoing general anesthesia: a network meta-analysis
Source: Front Neurol. 2025 Sep 19;16:1637566. doi: 10.3389/fneur.2025.1637566 (PMC12490997; doi:10.3389/fneur.2025.1637566)
Supplement: Supplementary file 1 [file Table_1.DOCX]

**Appendix 1 Search strategies**

**Pubmed**

| Search number | Query | Results |
| --- | --- | --- |
| 1 | (((Acupuncture Therapy[MeSH Terms]) OR (Electroacupuncture[MeSH Terms])) OR (Moxibustion[MeSH Terms])) OR (auricular acupuncture[MeSH Terms]) | 30964 |
| 2 | "abdominal needle"[Title/Abstract] OR "ACET acupoint catgut embedding therapy"[Title/Abstract] OR "acupoint catgut embedding"[Title/Abstract] OR "acupoint catgut embedding therapy"[Title/Abstract] OR "acupoint injection"[Title/Abstract] OR "Acupotom*"[Title/Abstract] OR "acupuncture"[Title/Abstract] OR "Acupuncture Therapy"[Title/Abstract] OR "Acupuncture Treatmen*"[Title/Abstract] OR "auricular acupunctur*"[Title/Abstract] OR "auriculo acupuncture"[Title/Abstract] OR "auriculoacupuncture"[Title/Abstract] OR "auriculotherapy"[Title/Abstract] OR "catgut embedding"[Title/Abstract] OR "catgut implantation"[Title/Abstract] OR "Ear Acupunctur*"[Title/Abstract] OR "earlobe acupuncture"[Title/Abstract] OR "electric acupuncture"[Title/Abstract] OR "electrical acupoint stimulation"[Title/Abstract] OR "electrical acupuncture"[Title/Abstract] OR "electro acupuncture"[Title/Abstract] OR "Electroacupuncture"[Title/Abstract] OR "electrode acupuncture"[Title/Abstract] OR "electronic acupuncture"[Title/Abstract] OR "Moxabustion"[Title/Abstract] OR "Moxibustion"[Title/Abstract] OR "Pharmacoacupuncture Therapy"[Title/Abstract] OR "Pharmacoacupuncture Treatment"[Title/Abstract] OR "shonishin"[Title/Abstract] OR "warming needle moxibustion"[Title/Abstract] | 35825 |
| 3 | (Postoperative Cognitive Complications[MeSH Terms]) OR (Emergence Delirium[MeSH Terms]) | 1533 |
| 4 | "Agitated Emergence"[Title/Abstract] OR "Emergence Agitation"[Title/Abstract] OR "Emergence Delirium"[Title/Abstract] OR "Emergence Excitement"[Title/Abstract] OR "post anaesthetic agitation"[Title/Abstract] OR "post anaesthetic delirium"[Title/Abstract] OR "post anaesthetic excitement"[Title/Abstract] OR "post anesthetic agitation"[Title/Abstract] OR "post anesthetic delirium"[Title/Abstract] OR "post anesthetic excitement"[Title/Abstract] OR "post operative cognitive complication"[Title/Abstract] OR "post operative cognitive decline"[Title/Abstract] OR "post operative cognitive deficit"[Title/Abstract] OR "post operative cognitive deterioration"[Title/Abstract] OR "post operative cognitive disorder"[Title/Abstract] OR "post operative cognitive dysfunction"[Title/Abstract] OR "post operative cognitive impairment"[Title/Abstract] OR "Post Operative Delirium"[Title/Abstract] OR "postanaesthetic agitation"[Title/Abstract] OR "postanaesthetic delirium"[Title/Abstract] OR "postanaesthetic excitement"[Title/Abstract] OR "postanesthetic agitation"[Title/Abstract] OR "postanesthetic delirium"[Title/Abstract] OR "postanesthetic excitement"[Title/Abstract] OR "Postoperative Cognitive Complication*"[Title/Abstract] OR "Postoperative Cognitive Decline"[Title/Abstract] OR "postoperative cognitive deficit"[Title/Abstract] OR "postoperative cognitive deterioration"[Title/Abstract] OR "Postoperative Cognitive Disorder*"[Title/Abstract] OR "Postoperative Cognitive Dysfunction"[Title/Abstract] OR "postoperative cognitive impairment"[Title/Abstract] OR "Postoperative Delirium"[Title/Abstract] OR "Postoperative Dementia*"[Title/Abstract] | 303,896 |
| 5 | (#1 OR #2) AND (#3 OR #4) | 81 |

**Embase**

| Search number | Query | Results |
| --- | --- | --- |
| 1 | 'acupuncture'/exp OR 'electroacupuncture'/exp OR 'moxibustion'/exp OR 'auricular acupuncture'/exp OR 'catgut embedding'/exp | 63025 |
| 2 | therapy':ti,ab,kw OR 'acupoint injection':ti,ab,kw OR 'acupotom*':ti,ab,kw OR 'acupuncture':ti,ab,kw OR 'acupuncture therapy':ti,ab,kw OR 'acupuncture treatmen*':ti,ab,kw OR 'auricular acupunctur*':ti,ab,kw OR 'auriculo acupuncture':ti,ab,kw OR 'auriculoacupuncture':ti,ab,kw OR 'auriculotherapy':ti,ab,kw OR 'catgut embedding':ti,ab,kw OR 'catgut implantation':ti,ab,kw OR 'ear acupunctur*':ti,ab,kw OR 'earlobe acupuncture':ti,ab,kw OR 'electric acupuncture':ti,ab,kw OR 'electrical acupoint stimulation':ti,ab,kw OR 'electrical acupuncture':ti,ab,kw OR 'electro acupuncture':ti,ab,kw OR 'electroacupuncture':ti,ab,kw OR 'electrode acupuncture':ti,ab,kw OR 'electronic acupuncture':ti,ab,kw OR 'moxabustion':ti,ab,kw OR 'moxibustion':ti,ab,kw OR 'pharmacoacupuncture therapy':ti,ab,kw OR 'pharmacoacupuncture treatment':ti,ab,kw OR 'shonishin':ti,ab,kw OR 'warming needle moxibustion':ti,ab,kw | 49172 |
| 3 | 'postoperative cognitive dysfunction'/exp OR 'emergence agitation'/exp | 3457 |
| 4 | 'agitated emergence':ti,ab,kw OR 'emergence agitation':ti,ab,kw OR 'emergence delirium':ti,ab,kw OR 'emergence excitement':ti,ab,kw OR 'post anaesthetic agitation':ti,ab,kw OR 'post anaesthetic delirium':ti,ab,kw OR 'post anaesthetic excitement':ti,ab,kw OR 'post anesthetic agitation':ti,ab,kw OR 'post anesthetic delirium':ti,ab,kw OR 'post anesthetic excitement':ti,ab,kw OR 'post operative cognitive complication':ti,ab,kw OR 'post operative cognitive decline':ti,ab,kw OR 'post operative cognitive deficit':ti,ab,kw OR 'post operative cognitive deterioration':ti,ab,kw OR 'post operative cognitive disorder':ti,ab,kw OR 'post operative cognitive dysfunction':ti,ab,kw OR 'post operative cognitive impairment':ti,ab,kw OR 'post operative delirium':ti,ab,kw OR 'postanaesthetic agitation':ti,ab,kw OR 'postanaesthetic delirium':ti,ab,kw OR 'postanaesthetic excitement':ti,ab,kw OR 'postanesthetic agitation':ti,ab,kw OR 'postanesthetic delirium':ti,ab,kw OR 'postanesthetic excitement':ti,ab,kw OR 'postoperative cognitive complication*':ti,ab,kw OR 'postoperative cognitive decline':ti,ab,kw OR 'postoperative cognitive deficit':ti,ab,kw OR 'postoperative cognitive deterioration':ti,ab,kw OR 'postoperative cognitive disorder*':ti,ab,kw OR 'postoperative cognitive dysfunction':ti,ab,kw OR 'postoperative cognitive impairment':ti,ab,kw OR 'postoperative delirium':ti,ab,kw OR 'postoperative dementia*':ti,ab,kw | 8857 |
| 5 | (#1 OR #2) AND (#3 OR #4) | 108 |

**Web of Science**

| Search number | Query | Results |
| --- | --- | --- |
| 1 | "TS=((abdominal needle) OR (ACET acupoint catgut embedding therapy) OR (acupoint catgut embedding) OR (acupoint catgut embedding therapy) OR (acupoint injection) OR (Acupotom*) OR (acupuncture) OR (Acupuncture Therapy) OR (Acupuncture Treatmen*) OR (auricular acupunctur*) OR (auriculo acupuncture) OR (auriculoacupuncture) OR (auriculotherapy) OR (catgut embedding) OR (catgut implantation) OR (Ear Acupunctur*) OR (earlobe acupuncture) OR (electric acupuncture) OR (electrical acupoint stimulation) OR (electrical acupuncture) OR (electro acupuncture) OR (Electroacupuncture) OR (electrode acupuncture) OR (electronic acupuncture) OR (Moxabustion) OR (Moxibustion) OR (Pharmacoacupuncture Therapy) OR (Pharmacoacupuncture Treatment) OR (shonishin) OR (warming needle moxibustion)) and Preprint Citation Index (Exclusion - Database) " | 137943 |
| 2 | "TS=((Agitated Emergence) OR (Emergence Agitation) OR (Emergence Delirium) OR (Emergence Excitement) OR (post anaesthetic agitation) OR (post anaesthetic delirium) OR (post anaesthetic excitement) OR (post anesthetic agitation) OR (post anesthetic delirium) OR (post anesthetic excitement) OR (post operative cognitive complication) OR (post operative cognitive decline) OR (post operative cognitive deficit) OR (post operative cognitive deterioration) OR (post operative cognitive disorder) OR (post operative cognitive dysfunction) OR (post operative cognitive impairment) OR (Post Operative Delirium) OR (postanaesthetic agitation) OR (postanaesthetic delirium) OR (postanaesthetic excitement) OR (postanesthetic agitation) OR (postanesthetic delirium) OR (postanesthetic excitement) OR (Postoperative Cognitive Complication*) OR (Postoperative Cognitive Decline) OR (postoperative cognitive deficit) OR (postoperative cognitive deterioration) OR (Postoperative Cognitive Disorder*) OR (Postoperative Cognitive Dysfunction) OR (postoperative cognitive impairment) OR (Postoperative Delirium) OR (Postoperative Dementia*)) and Preprint Citation Index (Exclusion - Database) " | 24831 |
| 3 | #1 AND #2 and Preprint Citation Index (Exclusion - Database) | 182 |

**Cochrane Library**

| Search number | Query | Results |
| --- | --- | --- |
| 1 | MeSH descriptor: [Acupuncture Therapy] explode all trees | 7247 |
| 2 | MeSH descriptor: [Electroacupuncture] explode all trees | 1193 |
| 3 | MeSH descriptor: [Moxibustion] explode all trees | 692 |
| 4 | MeSH descriptor: [Acupuncture, Ear] explode all trees | 269 |
| 5 | ('abdominal needle' OR 'ACET acupoint catgut embedding therapy' OR 'acupoint catgut embedding' OR 'acupoint catgut embedding therapy' OR 'acupoint injection' OR 'Acupotom*' OR 'acupuncture' OR 'Acupuncture Therapy' OR 'Acupuncture Treatmen*' OR 'auricular acupunctur*' OR 'auriculo acupuncture' OR 'auriculoacupuncture' OR 'auriculotherapy' OR 'catgut embedding' OR 'catgut implantation' OR 'Ear Acupunctur*' OR 'earlobe acupuncture' OR 'electric acupuncture' OR 'electrical acupoint stimulation' OR 'electrical acupuncture' OR 'electro acupuncture' OR 'Electroacupuncture' OR 'electrode acupuncture' OR 'electronic acupuncture' OR 'Moxabustion' OR 'Moxibustion' OR 'Pharmacoacupuncture Therapy' OR 'Pharmacoacupuncture Treatment' OR 'shonishin' OR 'warming needle moxibustion'):ti,ab,kw | 25297 |
| 6 | MeSH descriptor: [Postoperative Cognitive Complications] explode all trees | 134 |
| 7 | MeSH descriptor: [Emergence Delirium] explode all trees | 440 |
| 8 | ('Agitated Emergence' OR 'Emergence Agitation' OR 'Emergence Delirium' OR 'Emergence Excitement' OR 'post anaesthetic agitation' OR 'post anaesthetic delirium' OR 'post anaesthetic excitement' OR 'post anesthetic agitation' OR 'post anesthetic delirium' OR 'post anesthetic excitement' OR 'post operative cognitive complication' OR 'post operative cognitive decline' OR 'post operative cognitive deficit' OR 'post operative cognitive deterioration' OR 'post operative cognitive disorder' OR 'post operative cognitive dysfunction' OR 'post operative cognitive impairment' OR 'Post Operative Delirium' OR 'postanaesthetic agitation' OR 'postanaesthetic delirium' OR 'postanaesthetic excitement' OR 'postanesthetic agitation' OR 'postanesthetic delirium' OR 'postanesthetic excitement' OR 'Postoperative Cognitive Complication*' OR 'Postoperative Cognitive Decline' OR 'postoperative cognitive deficit' OR 'postoperative cognitive deterioration' OR 'Postoperative Cognitive Disorder*' OR 'Postoperative Cognitive Dysfunction' OR 'postoperative cognitive impairment' OR 'Postoperative Delirium' OR 'Postoperative Dementia*'):ti,ab,kw | 6515 |
| 9 | (#1 OR #2 OR #3 OR #4 OR #5) AND (#6 OR #7 OR #8) | 147 |

|  | 检索式 |
| --- | --- |
| 知网 | (主题：针灸 + 温针灸 + 电针 + 艾灸 + 耳穴 + 穴位埋线 + 穴位注射 + 火针 + 头针 + 穴位贴敷 + 皮内针 + 微针 + 针刺 + 脐灸 + 经皮穴位电刺激 + 体针 + 头皮针 + 腕踝针 + 激光针灸) AND （主题：术后认知功能） |
| 万方 | 主题:(针灸 OR 温针灸 OR 电针 OR 艾灸 OR 耳穴 OR 穴位埋线 OR 穴位注射 OR 火针 OR 头针 OR 穴位贴敷 OR 皮内针 OR 微针 OR 针刺 OR 脐灸 OR 经皮穴位电刺激) and 题名或关键词:(术后认知功能) |
| 维普 | [(任意字段=针灸 OR 任意字段=温针灸) OR 任意字段=电针) OR 任意字段=艾灸) OR 任意字段=耳穴) OR 任意字段=穴位埋线) OR 任意字段=穴位注射) OR 任意字段=火针) OR 任意字段=头针) OR 任意字段=穴位贴敷) OR 任意字段=皮内针) OR 任意字段=微针) OR 任意字段=针刺) OR 任意字段=脐灸) OR 任意字段=经皮穴位电刺激) OR 任意字段=体针) OR 任意字段=头皮针) OR 任意字段=腕踝针) OR 任意字段=激光针灸) AND 任意字段=术后认知功能)](https://qikan.cqvip.com/Qikan/search/index?LngMySearHistoryIdGuid=69f0b6c3-5f4f-4f06-9858-6eadb1de7657&from=Qikan_Article_History) |
| 中国生物医学 | ( "针灸"[全部字段:智能] OR "温针灸"[全部字段:智能] OR "电针"[全部字段:智能] OR "艾灸"[全部字段:智能] OR "耳穴"[全部字段:智能] OR "穴位埋线"[全部字段:智能] OR "穴位注射"[全部字段:智能] OR "火针"[全部字段:智能] OR "头针"[全部字段:智能] OR "穴位贴敷"[全部字段:智能] OR "皮内针"[全部字段:智能] OR "微针"[全部字段:智能] OR "针刺"[全部字段:智能] OR "脐灸"[全部字段:智能] OR "经皮穴位电刺激"[全部字段:智能] OR "体针"[全部字段:智能] OR "头皮针"[全部字段:智能] OR "腕踝针"[全部字段:智能] OR "激光针灸"[全部字段:智能]) AND "术后认知功能"[全部字段:智能] |
