# Supplementary figures and images for: The effects of different acupuncture modalities on postoperative cognitive function in elderly Chinese patients undergoing general anesthesia: a network meta-analysis
Source: Front Neurol. 2025 Sep 19;16:1637566. doi: 10.3389/fneur.2025.1637566 (PMC12490997; doi:10.3389/fneur.2025.1637566)

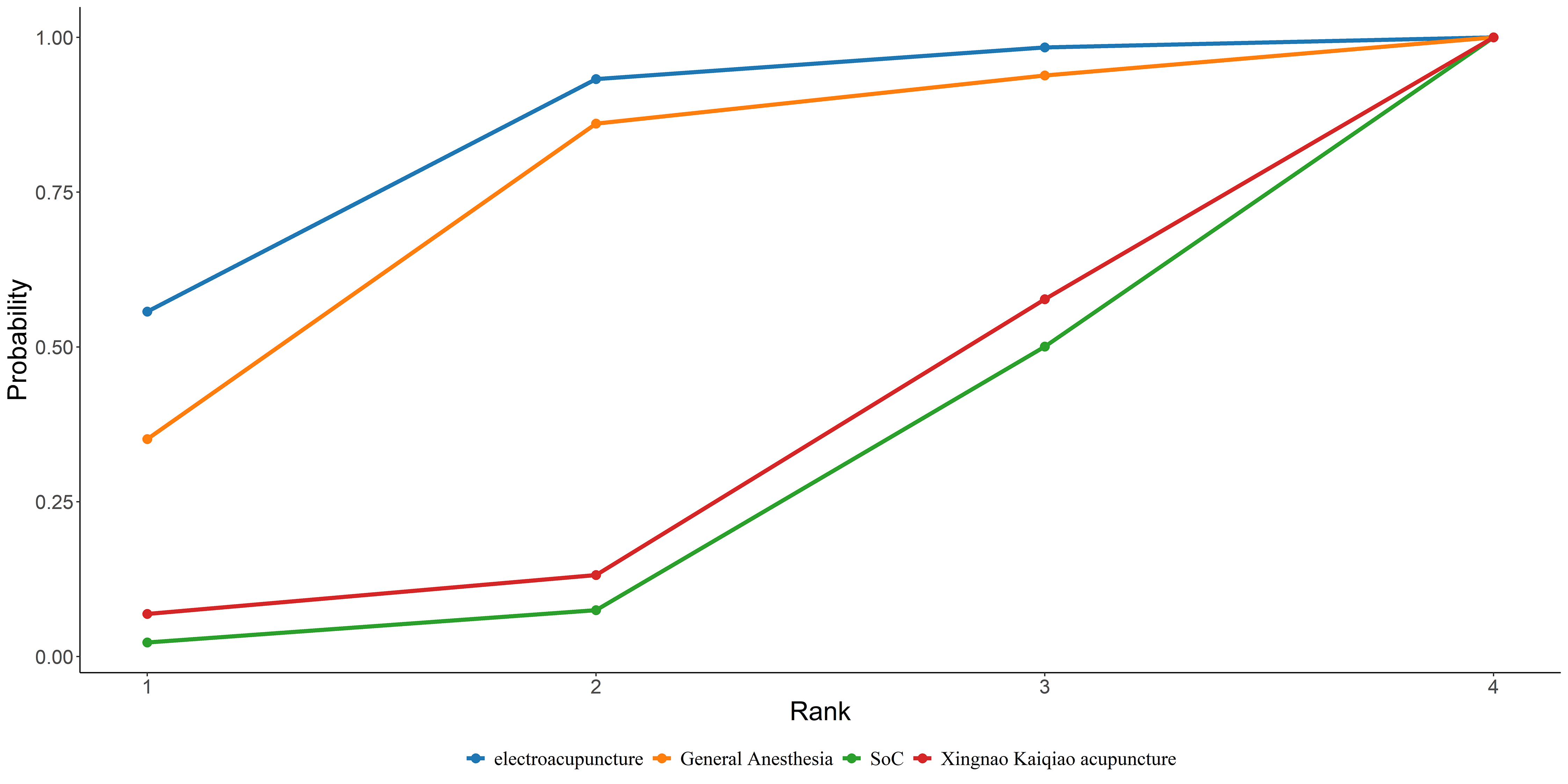

Supplement: Supplementary file 2 [file Image_1.TIF]

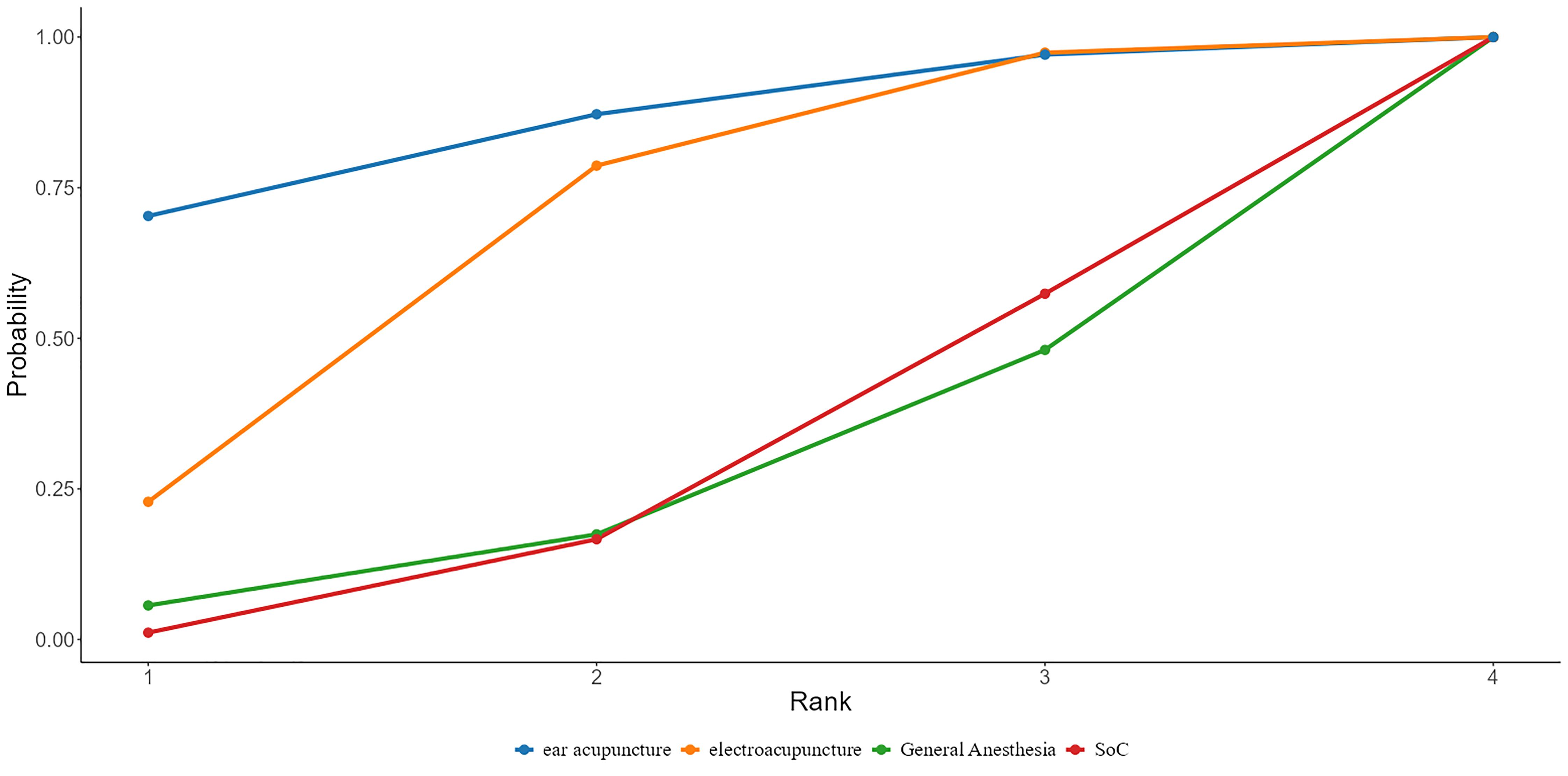

Supplement: Supplementary file 3 [file Image_2.TIF]
